# Preparation, *In-Vitro* Characterization and Pharmacokinetic Evaluation of Brij Decorated Doxorubicin Liposomes as a Potential Nanocarrier for Cancer Therapy

**Published:** 2018

**Authors:** Mohammadtaghi Fazel, Marjan Daeihamed, Mahraz Osouli, Ameneh Almasi, Azadeh Haeri, Simin Dadashzadeh

**Affiliations:** a *Department of Pharmaceutics, School of Pharmacy, Shahid Beheshti University of Medical Sciences, Tehran, Iran.*; b *Protein Technology Research Center, Shahid Beheshti University of Medical Sciences, Tehran, Iran. *; c *Pharmaceutical Sciences Research Center, Shahid Beheshti University of Medical Sciences, Tehran, Iran.*

**Keywords:** Liposomes, Pharmacokinetics, Brij, Doxorubicin, Stealth properties

## Abstract

The aim of current study was to investigate the effect of Brij decoration of liposomes on *in-vitro* and *in-vivo* characteristics of the nanocarriers. Two hydrophilic Brij surfactants (Brij 35 and Brij 78) with almost similar molecular weight but differing in acyl chain were incorporated into liposomal bilayers at two percentages (5% and 10%). Conventional liposomes (CL) containing egg phosphatidylcholine and cholesterol as well as Brij-enriched liposomal dispersions were prepared and characterized. *In-vivo* pharmacokinetics of various liposomal formulations and drug solution (six groups) was studied after intravenous administration to rats. Conventional and Brij enriched doxorubicin (DOX) liposomes had small size within 82-97 nm and showed homogenous distribution (PDI < 0.1). Drug encapsulation was higher than 97% in all liposomes. The drug release profiles proved sustained DOX release from various formulations. Based on the results of *in-vivo* studies, all five liposomes increased drug exposure and plasma concentration in comparison to free drug. However, DOX liposomes enriched with 5% of either Brij 35 or Brij 78 showed higher AUC values and lower clearance. Overall, Brij surfactants (5% of bilayer lipids) could be potentially used to improve liposomal pharmacokinetic parameters.

## Introduction

Liposomes are spherical self-assembling vesicles composed of phospholipids and cholesterol (CHOL) forming a lipid bilayer surrounding an aqueous core. Liposomes are by far the most promising nanoformulations for drug delivery due to numerous advantages such as biodegradability, biocompatibility, low toxicity, small particle size, composition versatility, drug solubilization, controlling drug release rate, improving drug pharmacokinetics, and targeting capability. Liposomes have been one of well-established nanocarriers for a wide variety of anticancer agents (1, 2). To be efficient in cancer therapy, the vesicle should be able to alter the pharmacokinetics and biodistribution of the encapsulated agents. The ability to protect cargoes from biodegradation and prolong their circulation time is crucial parameters for successful anticancer drug delivery nanosystems (3). Many tumors are characterized by the leaky vasculature and poor lymphatic drainage (4). Liposomes with a diameter < 200 nm and long circulation properties are too large to penetrate the barrier of normal healthy endothelium, but small enough to extravagate into the tumor tissues through the hyperpermeable leaky tumor blood vessels. These vesicles preferentially accumulate in the cancerous tissue via enhanced permeability and retention (EPR) effect and thereby achieve higher intratumoral drug concentration (5).

However, conventional liposomes hampered the early enthusiasm in liposomal drug delivery in cancer therapy due to predominant uptake by the reticuloendothelial system (RES) and inadequate physicochemical and plasma stability (6). Therefore, “stealth” coating of liposomes has been applied as an essential strategy to minimize opsonization and non-specific uptake by the RES, resulting in improved pharmacokinetic and biodistribution properties.

Polyethylene glycol (PEG) coating or PEGylation is the most common stealth engineering approach for *in-vivo* drug delivery of nanosystems. The ability of PEG coating to increase the blood circulation time and stealth properties of liposomes in order to enhance their tumor accumulation was a great achievement in cancer nanomedicine (6, 7).

However, high degree of carrier stabilization in the bloodstream and very long circulation half-life result in accumulation in the skin and in case of Doxil^®^ formulation lead to a cutaneous adverse event, known as the “hand and foot syndrome” (8). Upon vesicle accumulation in tumor tissue, the great stabilization also hampers the drug release in cancer tissue, interactions between vesicles and the surface of tumor cells, and internalization into the target cells (9). Moreover, PEG is not completely an inert material in biological systems as it has been found to induce production of PEG-specific antibodies and complement-mediated hypersensitivity reactions (10). Recent researches revealed that anti-PEG antibodies were detected in up to 25% of the healthy blood donors probably due to immune response caused by increased use of PEG in medicine, cosmetics, and industry. Hyper-production of anti-PEG antibodies has been reported in patients previously treated with PEG containing formulations (11), resulting in enhanced blood clearance especially upon repeated administration of PEGylated nanocarriers, so-called the accelerated blood clearance (ABC) phenomenon (12). These evidences stress the need to screen for potential alternatives for PEGylated liposomes.

Previous studies demonstrated that modification of liposomes with different polymers and surfactants, such as chitosan (13), hyaluronan (14, 15), and D-α-tocopheryl polyethylene glycol 1000 succinate (TPGS) (16) could efficiently protect liposomes in the circulation. Although these strategies have improved the liposomes performance in cancer treatment, there is still great need for further improvement of liposomal cancer therapeutics.

Brij^®^ molecules are single-chain surfactants, commercially available with different molecular weights, acyl chain structures, critical micelle concentrations (CMC), and hydrophilicity lipophilicity balance (HLB) values (17). Reversal of multiple drug resistance activities was also reported by Brij surfactants (18). It was hypothesized that Brij surfactants could have stealth nature and may replace the functions of distearoyl phosphoethanolamine-methoxy-poly (ethylene glycol)-2000 (DSPE-PEG2000) in the liposomal formulation. To investigate this hypothesis, in the current study, two hydrophilic Brij surfactants (Brij 35 and Brij 78) with similar molecular weight but differing in acyl chain (Table 1) were incorporated into the liposomal bilayers.

Anthracyclines, especially doxorubicin (DOX), are among the most active agents in cancer chemotherapy. DOX is most commonly used against a broad variety of solid and hematologic malignancies including breast, bladder, ovaries, stomach, lung, multiple myeloma, thyroid, soft tissue sarcoma, and Hodgkin′s lymphoma (19). 

**Figure 1:**
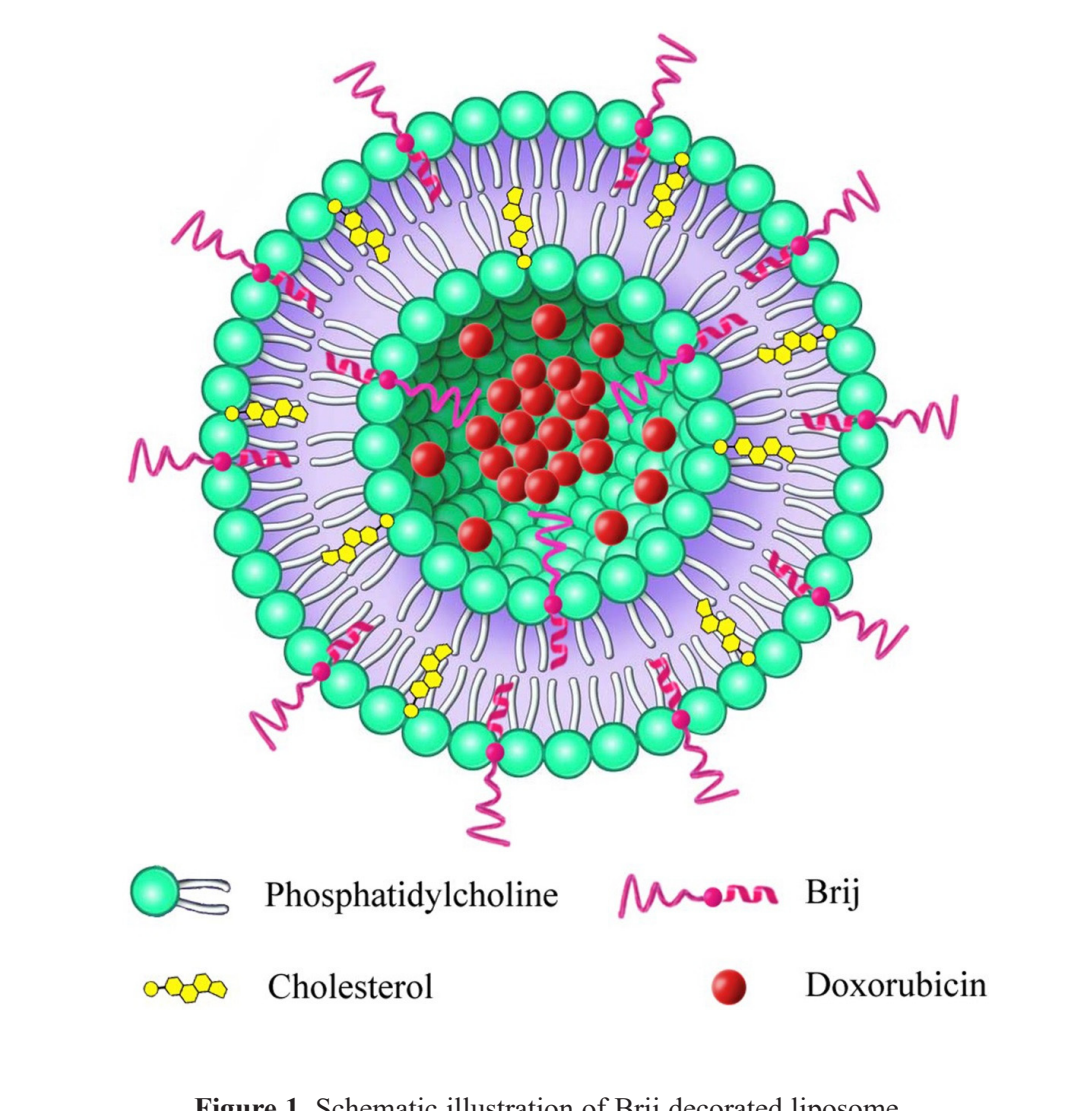
Schematic illustration of Brij decorated liposome

Taking into consideration the promising results of nanoliposomal drug delivery as well as DOX in cancer therapy, we attempted to develop a novel Brij-enriched DOX liposomal nanoformulation (Figure. 1) and study whether Brij type and percentage of incorporation affect plasma drug concentration profile. In this study, first we developed and characterized DOX liposomes enriched with 5% and 10% of either Brij 35 or Brij 78. Then, we evaluated pharmacokinetic properties of all formulations in Wistar rats. 

## Experimental

*Materials*

Pure egg phosphatidylcholine (EPC) was obtained from Lipoid GmbH (Ludwigshafen, Germany). CHOL (≥ 98% purity), Brij 78, and Brij 35 were purchased from Sigma-Aldrich (Germany). Doxorubicin hydrochloride (DOX) and daunorubicin hydrochloride were purchased from Pfizer (Germany). DOX stock solutions were stored in silanized glass vials at -20C. Chloroform, methanol, HPLC grade acetonitrile, methyl tert-butyl ether, potassium chloride, sodium chloride, potassium phosphate monobasic, sodium phosphate dibasic, ammonium thiocyanate, ferric chloride hexahydrate, and Triton X-100 were supplied by Merck (Darmstadt, Germany). Polycarbonate membrane filters were purchased from Northern Lipids Inc. (Vancouver, Canada). The dialysis membranes (12 kDa MWCO) were purchased from Spectrum Laboratories (Rancho Dominguez, CA, USA). All other chemicals were of analytical grade and used as received. 

*Liposome preparation*

The thin film hydration technique was used for liposome preparation. In brief, cholesterol, EPC, and Brij surfactants at desirable molar ratios (according to Table 2) were accurately weighed and dissolved in chloroform:methanol (3:1 v/v) in a round bottom flask and mixed thoroughly. The solvent was evaporated for about 30 min at 50 °C on the rotary evaporator. The thin lipid film was kept under reduced pressure for additional 3 h to remove all residual solvent. DOX was then remote-loaded using a transmembrane ammonium sulfate gradient method. The dried film was hydrated with 250 mM ammonium sulfate (pH 5.5) for 1 h. The resulting liposome dispersion was extruded through membranes with pore diameters of 200 nm, 100 nm, and 80 nm at 60 °C. To generate the ammonium sulfate gradient, the liposomal dispersions were dialyzed against phosphate-buffered saline (PBS, pH 7.4) for 5 h at room temperature to exchange the external buffer. The phospholipid concentration was determined by Stewart assay (20). For drug encapsulation, the liposomes were immediately incubated with DOX at the lipid to drug (L/D) molar ratio of 5 for 1 h at 60 C in dark. The un-encapsulated drug was separated by overnight dialysis against PBS (pH 7.4) at 4 °C.

*Encapsulation efficiency determination*

For encapsulation efficiency (EE) measurement, the liposome suspensions were separated from the free drug molecules. The collected liposomes were disrupted with 0.1% Triton X-100. DOX content in the liposomal samples was calculated using the calibration curve obtained from spectrophotometric analysis of the samples at 480 nm. %EE was calculated by the following equation:

%EE = (amount of drug in nanosystem after free drug removal /total amount of drug added in nanosystem) × 100.

*Particle size and distribution analysis*

The average size (Z-average size) and polydispersity index (PDI) of formulations were measured by dynamic light scattering (DLS) at 25 °C using a Zetasizer (Nano ZS, Malvern Instruments, UK). Liposomes were diluted to the appropriate volume with double distilled water before particle size analysis. 

*In-vitro drug release*

The *in-vitro* DOX release pattern from different liposomal formulations was investigated using dialysis bag method in PBS medium (21, 22). The samples (0.5 mL of liposomes or DOX solution) were transferred into a dialysis bag. The bag was then placed in 50 mL PBS. The release study was performed for 72 h at 37 °C with gentle shaking at 100 rpm and the vessels were protected from light. At predetermined time points, 2 mL aliquots were withdrawn from the beaker and replaced with the same volume of fresh PBS. At 8 h, 24 h, and 48 h, all buffered solutions outside the dialysis bag were replaced with fresh PBS. The DOX concentration was measured by fluorimetry using 470 nm excitation and 590 nm emission filters. To simulate *in-vivo* condition, in another set of experiments, the mixture of DOX formulations and human plasma (1:1 v/v) was placed in a dialysis bag. The drug release profile was evaluated by the same procedure.

*Stability studies in the presence of plasma*

The liposomal formulation (0.9 mL) was added to 0.1 mL of human plasma and the samples were incubated at 37 °C for 24 h with mild stirring before measurement. The adsorption of plasma protein on the vesicle surface was estimated by measuring the changes of the liposome size and PDI in the suspension. The size and PDI of samples were measured by light scattering as described before.

*Administration of liposomal formulations to rats*

The protocol for these animal studies was approved by the Ethical Committee of Shahid Beheshti Medical University. Male Wistar rats were purchased from the Pasteur Institute of Iran (Tehran, Iran), housed in cages at 22 ± 2 °C with a 12 h/12 h light/dark cycle. Animals had free access to rodent pellet diet and water and were allowed to acclimate for at least 1 week prior to the experiments.

The animals (220 ± 20 g) were randomly divided into six groups with 6 rats in each group. Drug dose in all animal groups was 2 mg/kg and was administered as intravenous (IV) injections into the tail vein. DOX solution and DOX loaded conventional liposomes (CL) were administered to two groups of these rats. Other animal groups received IV injections of either i) DOX loaded Brij 35 liposomes with 5% (Group 3) or 10% (Group 4) surfactant enrichment; or ii) DOX loaded Brij 78 liposomes containing 5% (Group 5) or 10% (Group 6) surfactant. Blood samples were drawn via tail vein at 0.083, 0.25, 0.5, 1, 2, 4, 6, 8, 10, and 24 h post-dosing and collected into EDTA-coated centrifuge tubes. The samples were centrifuged for 10 min at 5000 rpm and the plasma was stored at -20 °C for subsequent measurement of DOX within a week.

*Drug quantification in plasma samples*

DOX concentration in rat plasma was measured by a validated high-performance liquid chromatography (HPLC) method as described previously by our group (23, 24). The mobile phase consisted of water and acetonitrile (68:32, v/v; pH 2.6) was pumped at flow rate of 1.0 mL/min into the PerfectSil C18 column maintained at 35 °C. To 120 µL of plasma sample, 50 µL of daunorubicin hydrochloride solution (800 ng/mL in methanol) as the internal standard was added and vortex-mixed. The extraction of DOX was performed by adding 1 mL of a mixture of chloroform/methanol (4:1, v/v). After vortex mixing and centrifugation, the organic phase was collected, transferred to a clean tube, and evaporated to dryness under a stream of nitrogen. Dry residues were dissolved in 120 µL of the mobile phase and 100 µL of the sample was injected into the separation system. The column eluate was monitored with a fluorescence detector (with excitation at 470 nm and emission at 555 nm). It should be mentioned that total DOX concentration was measured in the samples.

*Pharmacokinetic data analysis*

The plasma concentration versus time profiles obtained following IV administration of various formulations were analyzed according to a non-compartmental pharmacokinetic model using PKSolver Microsoft Excel (25). Area under the curve from time zero to time infinity (AUC_0-inf_), area under the curve over 24 h (AUC_0-24_), mean residence time (MRT), total body clearance (Cl_T_), and volume of distribution at steady state (V_ss_) were calculated and compared.

*Statistical analysis*

All *in-vitro* experiments were repeated at least three times. For drug pharmacokinetic studies,* n*= 6 rats/group. All data are expressed as mean ± SD. The data were analyzed using Microsoft Excel and GraphPad Prism 6. A statistically significant difference was considered at *P* < 0.05 using Student′s t test and one-way analysis of variance (ANOVA) for two and multiple sample comparisons, respectively.

## Results and Discussion

*Preparation and characterization of conventional and Brij-enriched DOX loaded liposomes*

Liposomes were successfully prepared by the thin film hydration-extrusion method using EPC and CHOL as lipid materials at the molar ratio of 55:40. For drug loaded liposome preparation, the molar ratio of DOX to total bilayer forming materials was kept constant at 0.2. The bilayer composition, drug loading parameters, and physicochemical characterization of DOX liposomes were summarized in Table 2. The size of nanocarriers is of considerable importance in their *in-vivo* disposition. It has been previously reported that small sized (less than 100 nm) drug nanocarriers are plasma-stable and show prolong residence time in the bloodstream (26). In addition, the macromolecular drugs and small particles (less than 200 nm and preferably less than 100 nm) would benefit from the EPR effect driven tumor accumulation (27, 28). Our observations on DOX liposomal particle characteristics, showing highly homogenous vesicles (PDI < 0.1) within 82 - 97 nm size range (Table 2), reiterated the possibility of long plasma circulation time as well as efficient drug delivery to solid tumor. The size of all liposomes was tried to keep in a similar range in order to nullify the influence of this parameter on drug pharmacokinetic behavior.

All five liposomal formulations exhibited a similar EE (≥ 98%) (Table 2). The differences in EE or drug loading among the five formulations were not remarkable (*P* > 0.05) and Brij incorporation (even up to 10%) into bilayer did not influence drug loading. The obtained results were in agreement with our previous study that various DOX liposomes differing in size, lipid composition, L/D ratio, and surface charge showed efficient DOX loading (> 95%) (24). The high drug entrapment could be attributed to efficient sulfate gradient loading technique for DOX encapsulation into liposomes (29). 

*In-vitro drug release and plasma stability*

The release pattern of nanoparticles intended for IV administration is of prime importance. Drug loaded nanocarriers should reveal minimum cargo leakage in the blood circulation with controlled drug release at the target site. Rapid release of drug in the bloodstream is undesirable as it can lead to systemic toxicity (30, 31). Sustained DOX release from liposomal formulations is considered as a desirable release behavior.

To elucidate the *in-vitro* drug release behavior of free drug and conventional and Brij-enriched DOX loaded vesicles, we investigated the drug release dynamics in PBS (pH 7.4) at 37 °C by the dialysis method and fluorometric analysis. Release of free drug was very rapid with 48% and 91% drug released following 0.5 h and 1 h incubation, respectively. Under the same condition, the drug release from liposomal formulations was found to be very slow as less than 3.1% of the drug released in about 8 h for all formulations. After 8 h incubation in PBS, 10% Brij-enriched vesicles showed higher release percentages compared to the conventional formulation (*P* < 0.01).

**Figure 2:**
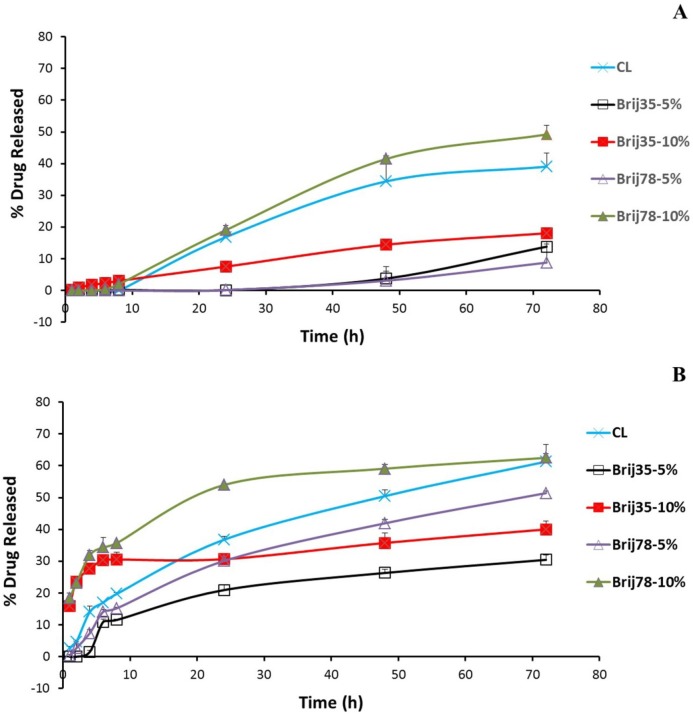
*In-vitro *release of DOX from conventional and Brij-enriched liposomes in PBS (A) and in the presence of plasma (B) at 37°C. Data are represented as mean ± SD (n = 3).

DOX release from liposomes occurred at least in three steps (Figure. 2). A lag time was observed in the first eight hour of the release study with <5% of the drug released. The phase lasted longer in 5% Brij-enriched formulations (both Brij35-5% and Brij78-5%). This phase may correspond to dissolution of insoluble DOX-sulfate in aqueous core of liposomes and DOX diffusion from inner core of the vesicles. Afterwards an almost faster DOX release rate was observed for about 60 h. In the third stage, the release rate slowed down again. This multi-stage release behavior could be attributed to the complexity of the involved processes such as dissolution of insoluble DOX-sulfate precipitate in the intraliposomal aqueous phase (32), drug partitioning into lipid bilayer, and DOX diffusion through the membrane (33).

**Table 1:** Physicochemical properties of Brij surfactants (17) used in this study for preparation of liposomes

**Trade Name**	**Structure**	**Acyl chain**	**Double bond**	**Molecular weight**	**Melting point (°C)**	**HLB** a	**CMC** b **(mM)**
Brij 35 (Brij L23)	C12H25(OCH2CH2)23OH	C12	0	1199	41–45	16.9	0.06
Brij 78 (Brij S20)	C18H37(OCH2CH2)20OH	C18	0	1152	44–46	15.3	0.006

a Hydrophilicity-Lipophilicity Balance.

b Critical Micelle Concentration.

**Table 2:** Compositions and physicochemical characterization of conventional and Brij-enriched DOX loaded liposomes. Data are represented as mean ± SD (n = 3).

Formulation	Liposome composition (molar ratio)	Symbol	%EE	Z-average (nm)	PDI
F1	EPC:CHOL (55:40)	CL	100.0 ± 1.3	94.0 ± 7.4	0.08 ± 0.05
F2	EPC:CHOL:Brij35 (55:40:5)	Brij35-5%	100.0 ± 1.0	88.6 ± 1.9	0.09 ± 0.04
F3	EPC:CHOL:Brij35 (55:40:10)	Brij35-10%	98.0 ± 1.0	82.1 ± 2.4	0.09 ± 0.01
F4	EPC:CHOL:Brij78 (55:40:5)	Brij78-5%	99.0 ± 1.4	95.2 ± 1.2	0.09 ± 0.02
F5	EPC:CHOL:Brij78 (55:40:10)	Brij78-10%	100.0 ± 1.0	96.5 ± 2.6	0.08 ± 0.04

**Table 3:** *In-vitro* plasma stability of conventional and Brij-enriched DOX loaded liposomes at 37  C. Date represented as mean ± SD (n = 3)

**Formulation**	**Z-average diameter (nm)**			**PDI**		
	**0**	**1h**	**24h**	**0**	**1h**	**24h**
CL	102.8 ± 1.5	121.5 ± 1.9	127.8 ± 2.1	0.17 ± 0.05	0.29 ± 0.05	0.21 ± 0.07
Brij35-5%	92.8 ± 0.3	93.3 ± 0.5	93.8 ± 0.4	0.19 ± 0.02	0.19 ± 0.01	0.20 ± 0.02
Brij35-10%	94.1 ± 0.5	94.3 ± 0.8	95.5 ± 0.9	0.25 ± 0.01	0.26 ± 0.02	0.25 ± 0.02
Brij78-5%	98.2 ± 0.4	98.9 ± 0.7	99.7 ± 0.7	0.21 ± 0.01	0.20 ± 0.01	0.23 ± 0.03
Brij78-10%	95.3 ± 0.5	96.1 ± 0.8	96.4 ± 0.8	0.24 ± 0.01	0.24 ± 0.02	0.25 ± 0.02

**Table 4:** Pharmacokinetic parameters of free DOX and conventional and Brij-enriched DOX loaded liposomes after IV administration to rats. Date represented as mean ± SD (n = 6).

**Formulation**	**AUC**_0-24_ (ng/ml*h)	**AUC**_0-inf_ (ng/ml*h)	**Cl**_T_ **(ml/h)**	**MRT**_0-24_ **(h)**	**C**_max_ **(ng/ml)**	**Vss** **(ml)**
DOX	635 ± 90	665 ± 83	669.4 ± 80.4	1.78 ± 0.54	381.8 ± 44.5	2443.6 ± 1098.7
CL	39683 ± 8914	42032 ± 8400	10.8 ± 1.9	3.37 ± 0.50	13431.8 ± 3598.5	72.8 ± 38.9
Brij35-5%	45682 ± 10519	72164 ± 10505##	6.2 ± 0.9##	3.21 ± 0.94	27585.7 ± 10857.9#	212.3 ± 129.0#
Brij35-10%	21905 ± 7222##	22109 ± 6947##	21.7 ± 7.2#	0.93 ± 0.27##	18776.4 ± 5394.4	50.6 ± 72.2
Brij78-5%	75501 ± 26619#	76988 ± 28399#	6.3 ± 1.9##	3.96 ± 0.78	17760.1 ± 2688.8	21.0 ±1 0.7#
Brij78-10%	33861 ± 3807	35457 ± 4256	12.4 ± 1.8	3.55 ± 0.48	10130.6 ± 1384.6	63.1 ± 7.7

# *P* value < 0.05 vs. CL,

## *P* value < 0.01 vs. CL.

**Figure 3:**
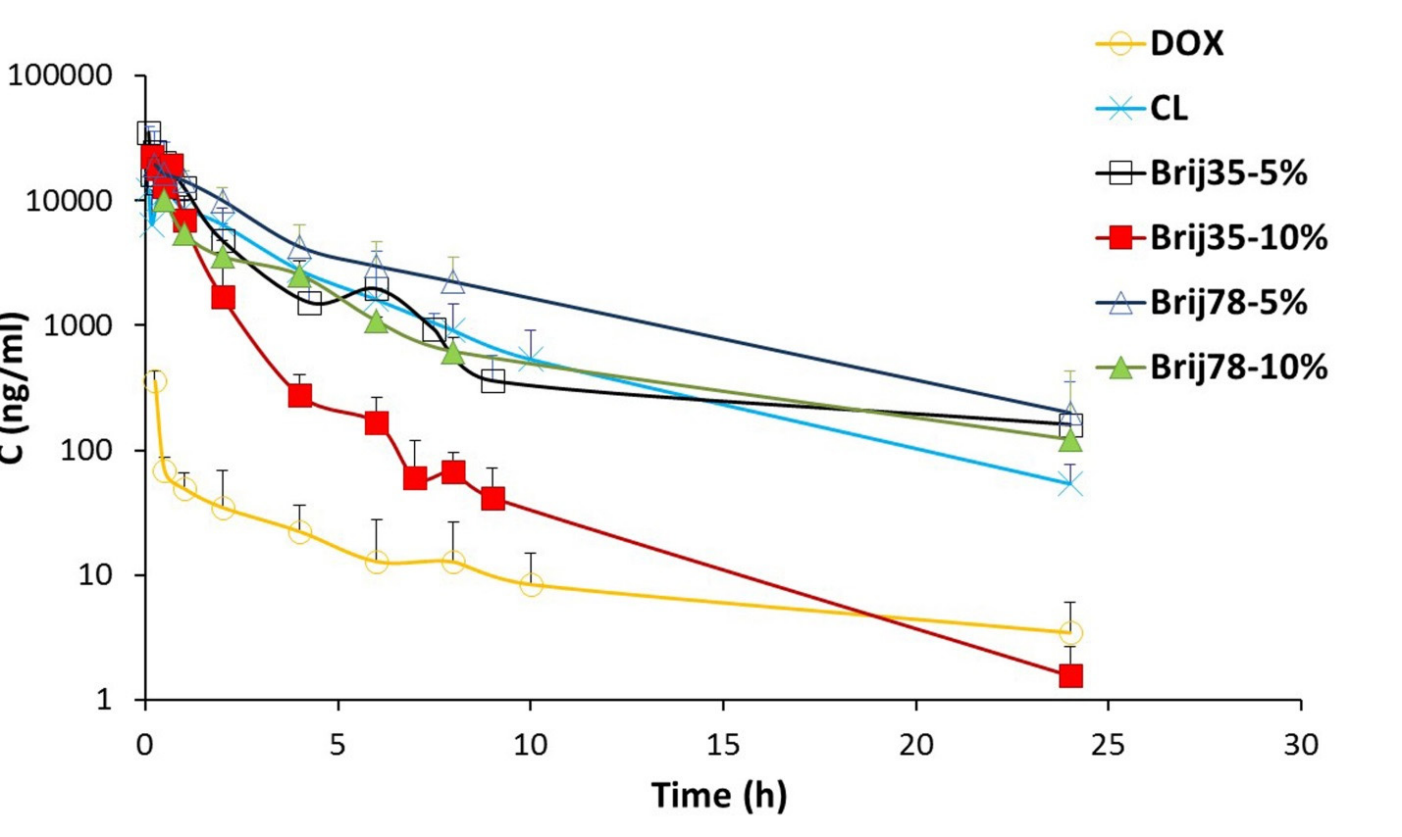
Mean plasma concentration of DOX (2 mg/kg) versus time curves obtained after IV administration of drug solution and conventional and Brij-enriched liposomes to rats. Data are represented as mean ± SD (n = 6)

The interactions between plasma proteins and liposomes are complex and can have a significant impact on the nanoparticles stability and their *in-vivo* fate. Serum lipoproteins can opsonize liposomes, destabilize their lipid bilayers leading to membrane disruption, loss of their contents, and enhancing their clearance (34). Thus, studying *in-vitro* release pattern and stability in the presence of plasma proteins is a fundamental issue for *in-vivo* applications of nanomaterials. 

To mimic the potential *in-vivo* release of DOX, *in-vitro* release was also studied in human plasma (Figure. 2B). After dialyzed for 24 h in the presence of plasma, the accumulative release percentages of CL, Brij35-10%, and Brij78-10% were achieved 36.8%, 30.7%, and 54.0%, respectively. However, 30.1% and only 20.9% of DOX released to medium from Brij78-5% and Brij35-5%, respectively (Figure. 2B). The Brij35-5% formulation showed the lowest release rate after incubation in plasma. 72 h incubation in the plasma resulted in 22%, 17%, 22%, 43%, and 13% increase in the percentages drug released for CL, Brij35-5%, Brij35-10%, Brij78-5%, and Brij78-10%, respectively (Figure. 2). Although *in-vitro* release studies in the presence of plasma try to mimic *in-vivo* condition, it may not exactly simulate the harsh *in-vivo* environment.

As shown in Table 3, the size and PDI of all Brij containing liposomes remained constant during 24 h incubation in plasma. However, immediately after adding plasma (time 0), the Z average diameter and the PDI of all samples were slightly higher than observed in the absence of plasma which may be due to presence of small protein particles in plasma. After 24 h of incubation of CL at 37 °C, a slight but statistically significant (*P* < 0.001) increase in the average particle size from 102.8 (± 1.5) nm to 127.8 (± 2.1) nm was observed (Table 3). At all time points, no evidence of particle aggregation or sedimentation was observed in any formulations. All enriched formulations revealed very good stability in plasma for a period of 24 h. In spite of increase in particle size of CL, the average particle size did not exceed 130 nm and therefore these vesicles could be considered as adequately stable.

The *in-vitro* release (Figure. 2B) and DLS (Table 3) results in the presence of plasma proved that the liposomes could have high integrity and stability in the physiological condition and are most likely good candidates to be used *in-vivo*.

*Pharmacokinetic study after intravenous injection in rats*

In this study, six different formulations of DOX (drug solution, CL, two Brij 35 containing liposomes and two Brij 78 containing liposomes) were administered to male rats as a single IV dose of 2 mg/kg. The effects of Brij type (Brij 35 and Brij 78) and Brij percentage (5% and 10%) on pharmacokinetic profile were investigated. The plasma concentrations of DOX after IV administration of six formulations are shown in Figure. 3. The pharmacokinetic parameters are summarized in Table 4.

At all time points, DOX plasma concentrations were higher for conventional as well as Brij-enriched liposomes when compared to drug solution (Figure. 3). Different DOX liposomes showed 34-119 times increase in AUC_0-24_ values and 27-72 times increase in C_max_ values compared with the control solution (Table 4). Cl_T_ of free DOX was at least 30 times higher than DOX liposomes. Contrary to DOX solution, elimination of DOX encapsulated in liposomes was slower and resulted in prolonged residence of drug in the bloodstream. In case of DOX liposomes, the extravasation is not as easy as for the free DOX molecules. Besides, DOX is not readily released from vesicles particularly within the first hours (Figure. 2) and therefore, the free DOX is not readily available for distribution. This led to lower V_ss_ with liposomes. Improvement of pharmacokinetic parameters by nanoencapsulation was reported by other researchers (35, 36). 

Drug AUC_0-inf _values after administration of different liposomes were in the following order: Brij78-5% > Brij35-5% > CL > Brij78-10% > Brij35-10% (Table 4). The clearance of Brij35-5% and Brij78-5% was significantly lower than those of conventional vesicles (*P *< 0.01). The increased AUC_0-inf _and decreased clearance of DOX in Brij35-5% and Brij78-5% groups are due to presence of adequate amount of Brij surfactants at the surface of liposomes. Stealth nature of Brij surfactants seems to prevent adsorption of plasma proteins and thereby avoids recognition by RES which ultimately leads to the enhanced drug exposure. Besides, adequate release rate should be considered as an influential parameter. In another study, enrichment of liposomes with 6.25 mol% TPGS enhanced systemic bioavailability and prolonged the circulation time of resveratrol following IV administration (16). Nag *et al*. reported the design of liposomes surface modified with 8 mol% of a novel acyl-anchored superhydrophilic polymer. The authors reported higher blood levels of modified liposomes after 24 h post-injection as compared with the plain vesicles (37).

Regarding both Brij 35 and Brij 78 enriched formulations, higher AUC values and lower clearance were observed for 5% enriched formulations compared to 10% enriched formulations (Table 4). These results are probably due to slower release of DOX from 5% enriched liposomes (Figure. 2). Zhuang *et al.* investigated the effects of chitosan coating with two concentrations of 0.3% and 0.6% on pharmacokinetic behavior of mitoxantrone liposomes (13). The liposomes coated with 0.3% chitosan solution showed the longer circulation time which was in agreement with lower release rate and higher stability of this formulation compared to vesicles coated with 0.6% chitosan (13).

Noticeably in the pharmacokinetic parameters, V_ss_ value of Brij78-5% was found to be 21 mL (the least among all studied groups) and almost equal to the total body water of rat (150 mL/kg) (16). This could lead to lower toxicity of this formulation.

It should be noted that higher AUC and C_max_ values and lower clearance achieved by 5% enriched formulations will be influential factors for improved efficacy but do not guarantee it. Therefore, efficacy studies in the tumor bearing animal model should be performed. 

## Conclusions

We have described the preparation and characterization of surface modified DOX liposomes by incorporation of 5-10% Brij surfactants (Brij 35 or Brij 78) into the vesicle bilayers. The size, PDI, drug loading, release behavior, and stability of liposomes were suitable for successful drug delivery. *In-vivo* experiments clearly revealed that the 5% enriched formulations (Brij35-5% and Brij78-5%) improved the pharmacokinetic properties of DOX compared to CL. The Brij35-5% and Brij78-5% remarkably increased AUC and decreased drug clearance. It appears that Brij-enriched DOX liposomes hold great promise for cancer therapy. The results presented in this investigation open the way for further researches to explore the potential improvement in cytotoxicity and anti-tumor activities by this novel delivery system.
